# Interrelationships and determinants of aging biomarkers in cord blood

**DOI:** 10.1186/s12967-022-03541-1

**Published:** 2022-08-09

**Authors:** Brigitte Reimann, Dries S. Martens, Congrong Wang, Akram Ghantous, Zdenko Herceg, Michelle Plusquin, Tim S. Nawrot

**Affiliations:** 1 Centre for Environmental Sciences, Hasselt University, Hasselt, Belgium; 2 Epigenomics and Mechanisms Branch, International Agency for Research On Cancer (IARC), Lyon, France; 3 School of Public Health, Occupational and Environmental Medicine, KU Leuven, Leuven, Belgium

**Keywords:** DNAm age; telomere length; mitochondrial DNA content, Global methylation, Aging biomarkers, Cord blood, ENVIR*ON*AGE cohort

## Abstract

**Background:**

Increasing evidence supports the concept of prenatal programming as an early factor in the aging process. DNA methylation age (DNAm age), global genome-wide DNA methylation (global methylation), telomere length (TL), and mitochondrial DNA content (mtDNA content) have independently been shown to be markers of aging, but their interrelationship and determinants at birth remain uncertain.

**Methods:**

We assessed the inter-correlation between the aging biomarkers DNAm age, global methylation, TL and mtDNA content using Pearson's correlation in 190 cord blood samples of the ENVIR*ON*AGE birth cohort. TL and mtDNA content was measured via qPCR, while the DNA methylome was determined using the human 450K methylation Illumina microarray. Subsequently, DNAm age was calculated according to Horvath's epigenetic clock, and mean global, promoter, gene-body, and intergenic DNA methylation were determined. Path analysis, a form of structural equation modeling, was performed to disentangle the complex causal relationships among the aging biomarkers and their potential determinants.

**Results:**

DNAm age was inversely correlated with global methylation (r = -0.64, p < 0.001) and mtDNA content (r = − 0.16, p = 0.027). Cord blood TL was correlated with mtDNA content (r = 0.26, p < 0.001) but not with global methylation or DNAm age. Path analysis showed the strongest effect for global methylation on DNAm age with a decrease of 0.64 standard deviations (SD) in DNAm age for each SD (0.01%) increase in global methylation (p < 0.001). Among the applied covariates, newborn sex and season of delivery were the strongest determinants of aging biomarkers.

**Conclusions:**

We provide insight into molecular aging signatures at the start of life, including their interrelations and determinants, showing that cord blood DNAm age is inversely associated with global methylation and mtDNA content but not with newborn telomere length. Our findings demonstrate that cord blood TL and DNAm age relate to different pathways/mechanisms of biological aging and can be influenced by environmental factors already at the start of life. These findings are relevant for understanding fetal programming and for the early prevention of noncommunicable diseases.

**Graphical Abstract:**

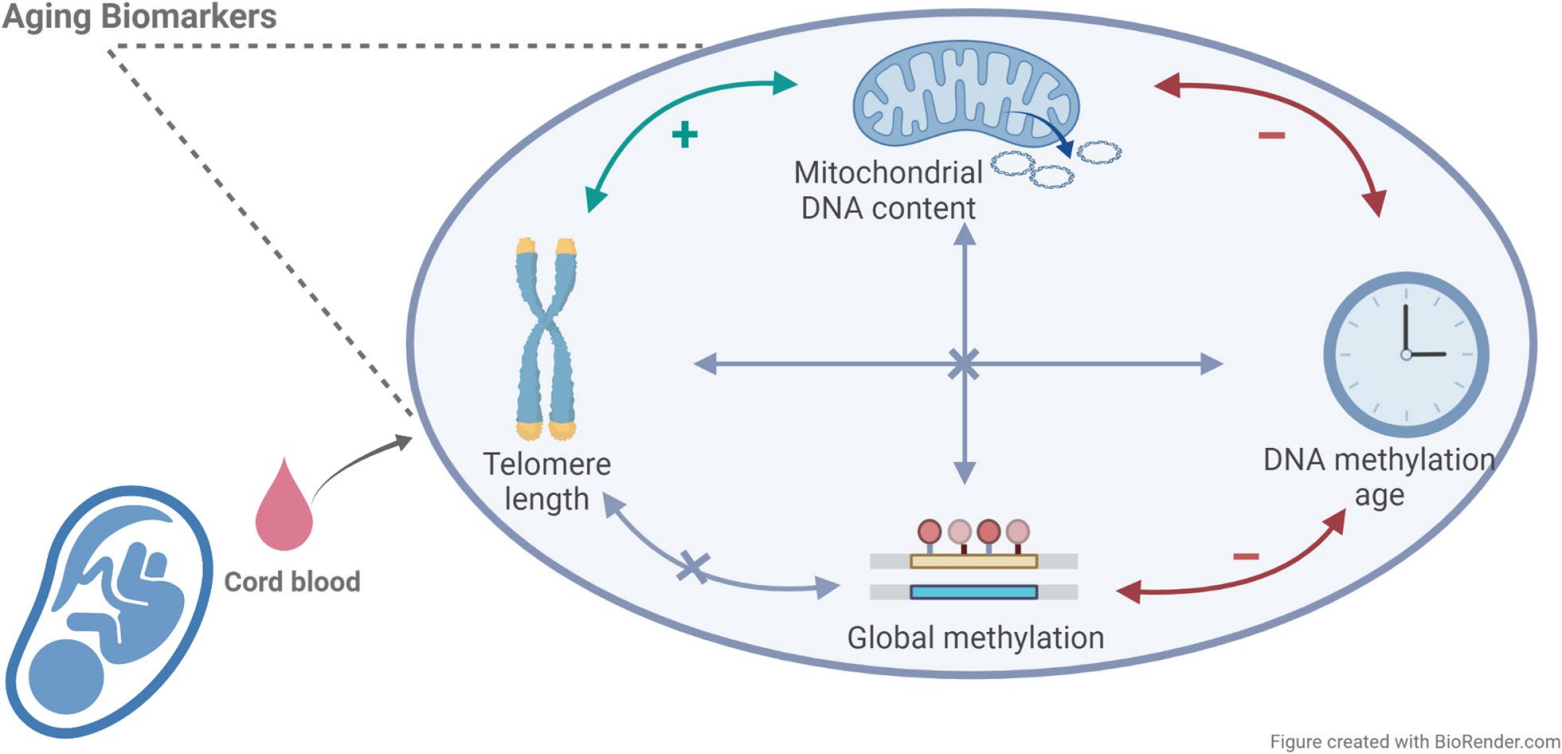

**Supplementary Information:**

The online version contains supplementary material available at 10.1186/s12967-022-03541-1.

## Background

Aging starts at conception; even before conception, environmental factors can prime its conditions. It is commonly accepted that genetic make-up and environment are major determinants of healthy aging and life expectancy [1, 2]. However, evidence is accumulating that the rate of age-associated functional decline is also determined by prenatal programming [3–7]. DNA methylation age (DNAm age), global genome-wide DNA methylation, telomere length (TL), and mitochondrial DNA content (mtDNA content) have independently been reported to correlate with chronological age and, therefore, these markers have been employed as potential measures of aging [8, 9]. DNAm age has been indicated as an epigenetic clock with biological significance in the context of age acceleration (AA), which has previously been linked to obesity [10], age-related diseases [11–13], and all-cause mortality [11, 14, 15].

The mechanisms by which global DNA hypomethylation contributes to the process of aging and age-related noncommunicable diseases (NCD) are not yet well understood. Different modes of action have been suggested, such as the increase of genomic instability through the accumulation of DNA damage-induced chromatin modifications [16, 17] or decreased efficacy of DNA (cytosine-5)-methyltransferase 1 (DNMT1) [18]. A decreased mtDNA copy number in peripheral blood has been linked with aging and mortality [19, 20]. TL is variable at birth, tracks over the lifetime [21], and decreases with advancing age. Furthermore, TL has consistently been linked to cellular senescence and disease susceptibility [8, 22]. According to the TL/mitochondrial axis of aging [23], reactive oxygen and nitrogen species, together with other free radicals, target telomeres in an age-dependent manner. Dysfunctional telomeres can lead to decreased mitochondrial biogenesis and function via repression of Pgc-1α,β and Sirt1 gene expression [24, 25], causing an age-related decrease in mtDNA content and general health [19, 26]. Intriguingly, this process may even start before birth, when newborn telomeres are influenced by the in utero environment [4, 5] and impact the entire life course [6, 21]. DNA methylation status, measured as global methylation, DNAm age, TL, and mtDNA content at birth, could have important implications for overall life expectancy and disease susceptibility later in life [23].

**Fig. 1:**
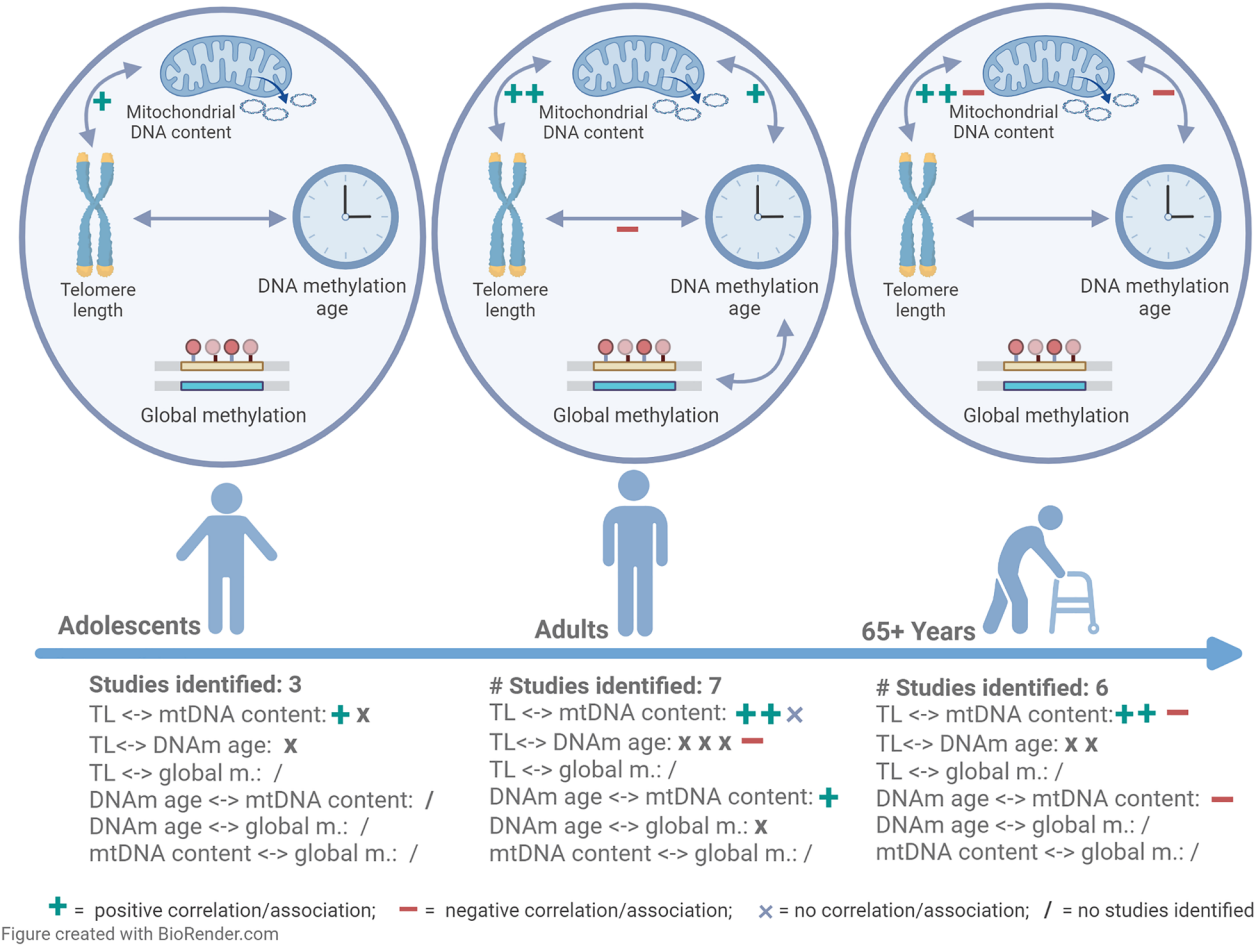
Overview of the identified studies investigating the interrelationship between the aging biomarkers telomere length (TL)), DNA methylation age or DNA methylation age acceleration (DNAm age/DNAm AA), mitochondrial DNA content (mtDNA content) or global DNA methylation (global m.) in the age groups of adolescents (mean age ≥ 10), adults (mean age > 19) and older people (mean age > 65). The arrows in the ovals show which correlations/associations have been investigated in the different age groups

Various studies dealt with the relationship between telomere length and other individual aging biomarkers in the elderly, adults, and adolescents [27–41] (Fig. 1 and Additional file 1: Table S1). To unravel the relationships between the different aging biomarkers already in cord blood and confirm or disprove previous findings in older age groups is important because these findings provide evidence for the starting point of biological mechanisms leading to age-related disease. In the present study, we first investigated the inter-correlations between aging biomarkers to obtain insights into the underlying aging mechanisms in the ENVIR*ON*AGE (ENVIRonmental influence *ON* AGEing in early life) birth cohort. In a second step, we compared the effects of potentially important determinants of early-life aging.

## Methods

### Study population

**Fig. 2:**
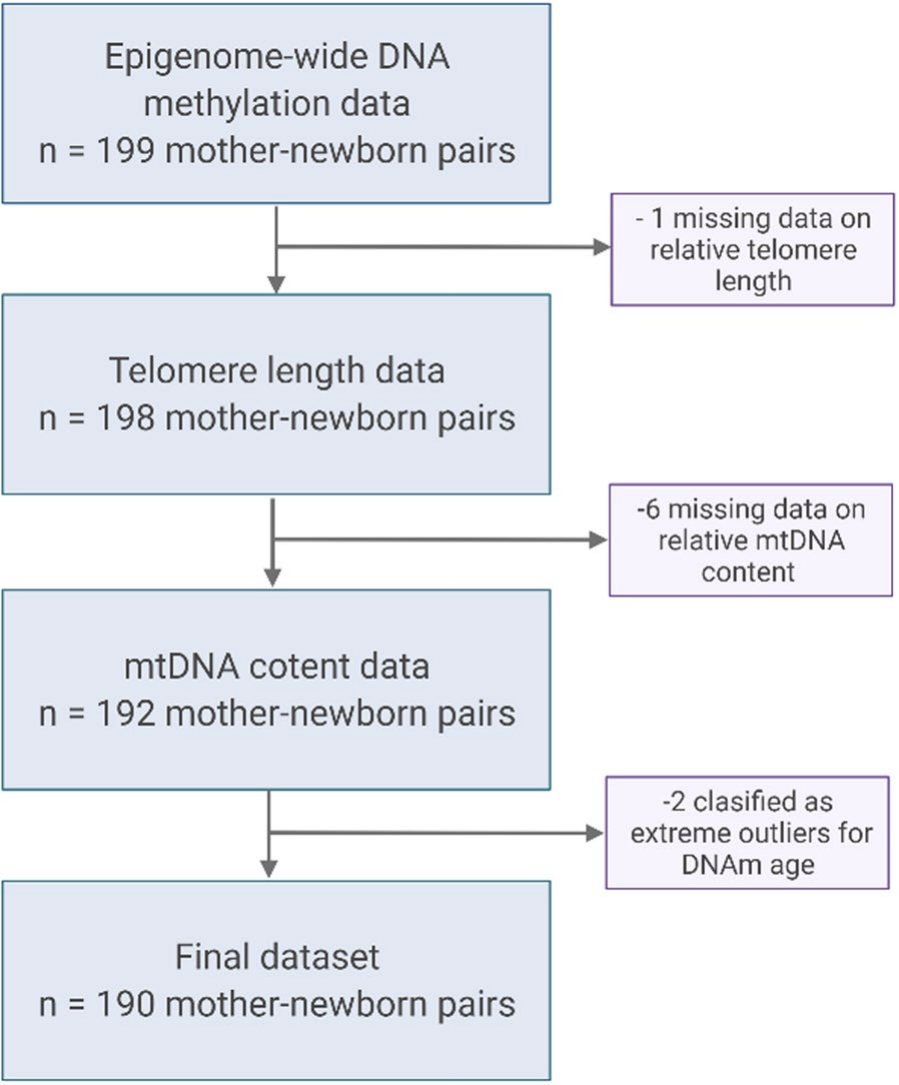
Flow chart visualizing the sample selection. Initially, 199 mother-newborn pairs participating in the ENVIR*ON*AGE birth cohort between July 2014 and June 2015, with epigenome-wide DNA methylation data, were selected. The final number of participating mother-newborn pairs included in the analysis was 190

Our study initially enrolled 199 eligible mother-newborn pairs with singleton newborns in the ENVIR*ON*AGE birth cohort [42]. These mother-newborn pairs were recruited as a subset of the ongoing prospective cohort between July 2014 and June 2015 at the East-Limburg Hospital in Genk, Belgium. This study was conducted according to the principles outlined in the Helsinki Declaration [43] after approval by the Ethical Committee of Hasselt University and the East-Limburg Hospital in Genk. Written informed consent was obtained from all participating mothers at recruitment. Epigenome-wide methylation status of the CpG sites was retrieved from cord blood samples in the framework of the EXPOsOMICS project (FP7) [44]. Data on relative TL were available for 198 neonates. For six of these neonates’ data on mtDNA content was missing. Finally, two samples were removed from the analysis because they were classified as extreme outliers concerning the calculated DNAm age [> 3 × interquartile range (IQR) below the first quartile or above the third quartile], using the R package NCmisc 1.1.6. Therefore, the final sample size in this study was 190 (Fig. 2).

Maternal body mass index (BMI) was determined during the first antenatal visit (weeks 7–9 of pregnancy) by dividing weight in kilograms by height in meters squared. The conception date was estimated based on the first ultrasonographic examination. After delivery, mothers filled out a study questionnaire, which collected detailed information about sociodemographic and lifestyle factors for both mothers and fathers. Paternal age was missing in nine cases and imputed with the mean paternal age at birth. Parity was classified into three categories for mothers having their first, second, and third or more newborns. Maternal educational level was coded as “low" for mothers who did not obtain any diploma, “middle” when they obtained a high school diploma, and “high” when they obtained a college or university degree. Maternal smoking status was categorized as “never smoker,” when the mother never smoked before or during pregnancy, “former smoker” when the mother had quit smoking before pregnancy, and “smoker” when the mother continued to smoke during pregnancy. Newborn ethnicity was categorized based on the grandparents' origin and was classified as European when two or more grandparents were European and non-European when at least three grandparents were of non-European origin. The season of delivery was divided into the cold season (October 1—March 31) and the warm season (April 1—September 30).

### Cord blood sample collection

Cord blood samples were collected directly after delivery in BD Vacutainer^®^ Lithium Heparin, Plus Plastic K2EDTA Tubes (BD, Franklin Lakes, NJ, USA) and centrifuged at 3200 rpm for 15 min. After that, buffy coat and plasma were separated and frozen instantly at − 80 °C.

### Epigenome-wide DNA methylation

Cord blood DNA was extracted and processed at the Epigenomics and Mechanisms Branch (formerly Epigenetics Group), International Agency for Research on Cancer (IARC). In detail, after thawing and extraction with the QIAamp DNA mini Kit (Qiagen Ltd, Manchester, UK), DNA was first bisulfite-converted using the Zymo EZ DNA methylation^™^ kit (Zymo, Irvine, CA, USA), consequently hybridized to Illumina Infinium Human Methylation 450K BeadChip arrays [45] and scanned using the Illumina HiScanSQ system. After background subtraction with Illumina GenomeStudio, the raw intensity data were preprocessed, including the calculation of the methylation level at each CpG as the beta-value, the normalization using the funnorm normalization of the minfi package [46], and quality control employing in-house software within the R statistical computing environment. Samples underwent further quality control employing Illumina’s detection *p*-value > 0.05 and bead count lower than 3, excluding failed samples. Additionally, background subtraction and dye bias correction were performed on Infinium II probes. Finally, data were also trimmed for outliers containing values more than three interquartile ranges below the first quartile or above the third quartile so that 485,512 probes remained for analysis.

### Mean relative TL and mtDNA content measurements

The quantity and purity of the extracted DNA were assessed by spectrometric analysis using the Nanodrop 1000 spectrophotometer (Isogen, Life Science, Belgium), and integrity was evaluated using agarose gel electrophoresis. All measurements were performed in triplicate on a 7900HT Fast Real-Time PCR System (Applied Biosystems, Foster City, CA, USA) using a 384-well format. DNA quantity was determined through the Quant-iT™ PicoGreen® dsDNA Assay Kit (LifeTechnologies, Europe), to ensure a uniform DNA input of 5 ng/PCR reaction. Average relative telomere length and mtDNA content were measured by a modified quantitative real-time PCR (qPCR) protocol as described previously [5, 47, 48] and in detail provided in Additional file 2: Text S1. PCR cycles are described in detail in Additional file 3: Tables S2–S4. Telomere assay‑precision expressed by the intra-class correlation coefficient (ICC) [49] was 0.936 (95% CI: 0.808 to 0.969) for the inter-assay ICC and 0.952 (95% CI: 0.947 to 0.956) for the intra-assay ICC was. Cycle thresholds of the telomere and mtDNA amplifications were normalized relative to the cycle thresholds of the single-copy gene amplifications Additional file 3: Tables S2–S4 using the qBase software (Biogazelle, Zwijnaarde, Belgium). Relative average telomere lengths and mitochondrial content were expressed as the individual relative ratio to the average ratios of the entire sample set.

### Statistics

DNAm age was calculated according to the epigenetic clock developed by Horvath using the Bioconductor package “methylclock” [50] with the cell count reference option according to Bakulski [51]. The degree of methylation was expressed as the percentage of methylated cytosines over the sum of methylated and unmethylated cytosines. Global DNA methylation was calculated by calculating the study population's arithmetic mean of the beta-values (epigenome-wide average DNA methylation). Additionally, mean methylation of the three functional gene regions, promoter (n = 140,003 probes), gene body (n = 158,210 probes), and intergenic regions (n = 187,299 probes) was calculated. TL and mtDNA were log_10_ transformed to improve normal distribution.

In the statistical analysis, Pearson correlation was applied to address the relationship between the four aging biomarkers. In the next step, path analysis, adjusted for *a priori* selected covariates, was performed to assess the associations between the aging biomarkers and establish significant early-life aging determinants. This form of structural equation modeling (SEM) is characterized by multiple linear regression equations with simultaneous estimation of regression coefficients for all hypothesized relations between the variables. The path analysis model included sex, gestational age newborn ethnicity, birthweight, maternal smoking, maternal education, maternal early-pregnancy BMI, and parity as covariates. For TL and mtDNA content, additionally, white blood cell count, season of delivery, and parental age, and for global methylation cell-type distribution according to Bakulski [51] were included as covariates. The presence of random effects was taken into account by using (i) the residuals of DNAm age and global methylation regressed on array chip and array position and (ii) the residuals of TL and mtDNA content regressed on sample storage time. The following assumptions regarding the direction of the associations were made: (i) TL affected mtDNA content and was in turn affected by DNAm age and global methylation, (ii) DNAm age was affected by mtDNA content and global methylation, (iii) global methylation was affected by mtDNA content. The path analysis was accomplished with the lavaan package, version 0.6–5 [52]. Statistical significance was defined as p < 0.05. All data analyses were performed in RStudio using R 3.5.2.

## Results

### Demographics

Demographic characteristics and perinatal factors of the mother-newborn pairs are reported in Table 1. The newborns in this study were mostly of European origin (89%) with a mean (SD) gestational age of 39.15 (± 1.54) weeks and a mean (SD) birthweight of 3393 (± 484) g. The TL and mtDNA content range was between 0.51–1.58 and 0.25–4.35, respectively, and the mean (± SD) DNAm age was 0.46 (± 0.26) years. The mothers were on average 29 (± 4.5) years old and had a pre-pregnancy BMI of 24.39 (± 4.34) kg/m^2^.

**Table 1:** Population characteristics and perinatal factors from n = 190 participants

Characteristics	Mean (± SD) or n (%)
Newborn	
Girls, n	88 (49.2%)
Birthweight, grams	3393.08 ± 484.49
European, n	169 (89.0%)
Gestational age, weeks	39.15 ± 1.54
Relative telomere length	0.95 ± 0.16 [0.51–1.58]
Relative mtDNA content	1.04 ± 0.47 [0.25–4.35]
DNAm age, years	0.46 ± 0.26 [-0.23–1.21]
Global DNA methylation, proportion	0.50 ± 10 [47–53]
Gene-promotor methylation, proportion	0.29 ± 0.01 [0.27–0.31]
Gene-body methylation, proportion	0.64 ± 0.01 [0.60–0.67]
Intergenic-region methylation, proportion	0.52 ± 0.01 [0.49–0.55]
Maternal	
Age, years	29.35 ± 4.5
Early pregnancy BMI, kg/m2	24.39 ± 4.34
Education	
Low, n	27 (14.2%)
Middle, n	66 (34.7%)
High, n	97 (51.1%)
Smoking, n	
Never smoked	123 (64.7%)
Former smoker	44 (23.2%)
Smoked during pregnancy	23 (12.1%)
Parity	
1, n	104 (54.7%)
2, n	58 (30.5%)
≥ 3, n	28 (14.7%)
Paternal age, years	31.81 ± 5.34
Season of delivery	
October 1–March 31	84 (44.2%)
April 1—September 30	106 (55.8%)

The numbers represent counts (percentages) for categorical and means (± standard deviation) for continuous variables. For TL, mtDNA content, and methylation values, additionally, the range is reported as between brackets [lowest value—highest value]

*TL =* relative telomere length, *DNAm age =* epigenetic age

### Correlation between the biomarkers of aging

**Fig. 3:**
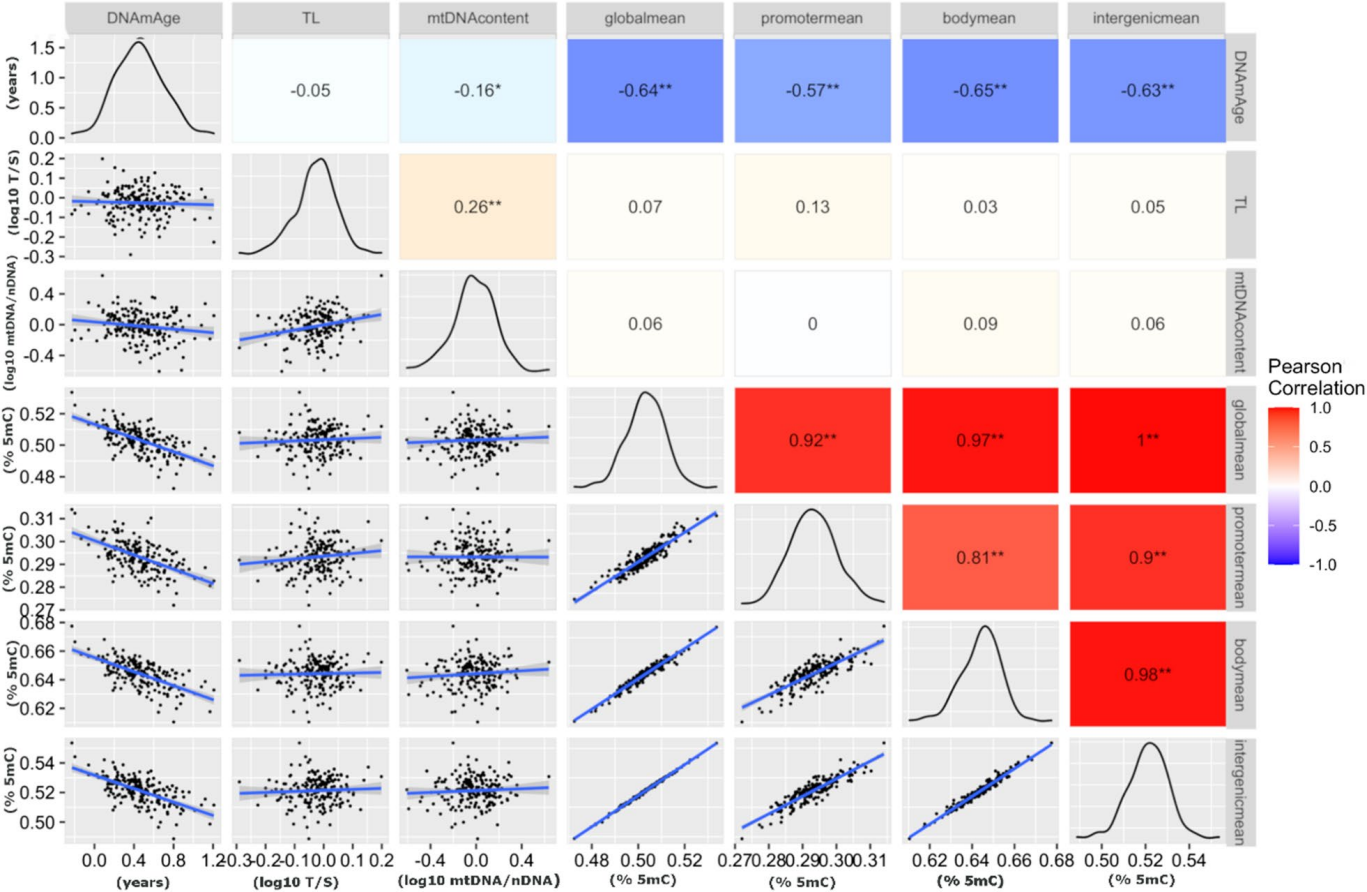
Pearson correlations between DNAm Age = epigenetic age calculated according to Horvath [9], TL = relative telomere length, mtDNAcontent = relative mitochondrial DNA content, globalmean = mean global DNA methylation, promotermean = mean methylation of the promoter gene-region, bodymean = mean methylation of the gene-body and intergenicmean = mean methylation of the intergenic region. In the top right corner, the correlation coefficients, and in the bottom left corner scatterplots of the correlation with regression line and 0.95% confidence interval are shown. The diagonal density plots display the distribution of observations. 5mC = 5-methylcytosine; mtDNA = mitochondrial DNA; nDNA = nuclear DNA; T/S = telomere/ single copy gene ratio * p < 0.05; ** p < 0.001

Figure 3 shows the inter-correlation of the different biological markers of aging. Cord blood DNAm age showed a significant, inverse correlation with all other aging biomarkers, except with TL where the correlation was not significant. The strength of correlation for DNAm age was in descending order with cord blood mean (i) gene body methylation, r = − 0.65, (ii) global methylation r = − 0.64, (iii) intergenic region methylation r = − 0.63 and (iv) promoter methylation r = − 0.57 (all p < 0.001) and with mtDNA content (r = − 0.16, p = 0.027). Cord blood TL and mtDNA content were significantly correlated (r = 0.26, p < 0.001). Global methylation was significantly correlated with promoter, gene body and intergenic region methylation (r = 0.92, r = 0.97 and r = 1, all p < 0.001).

When stratified for sex, these correlations remained significant (Additional file 4: Figure S1 and Additional file 5: Figure S2). For newborn girls, the correlation between TL with mean global, body, intergenic methylation, and DNAm age changed direction, as did the correlation between mtDNA content and mean promoter methylation in boys (all remaining non-significant).

### Associations between the aging biomarkers and covariates in the path analysis

**Fig. 4:**
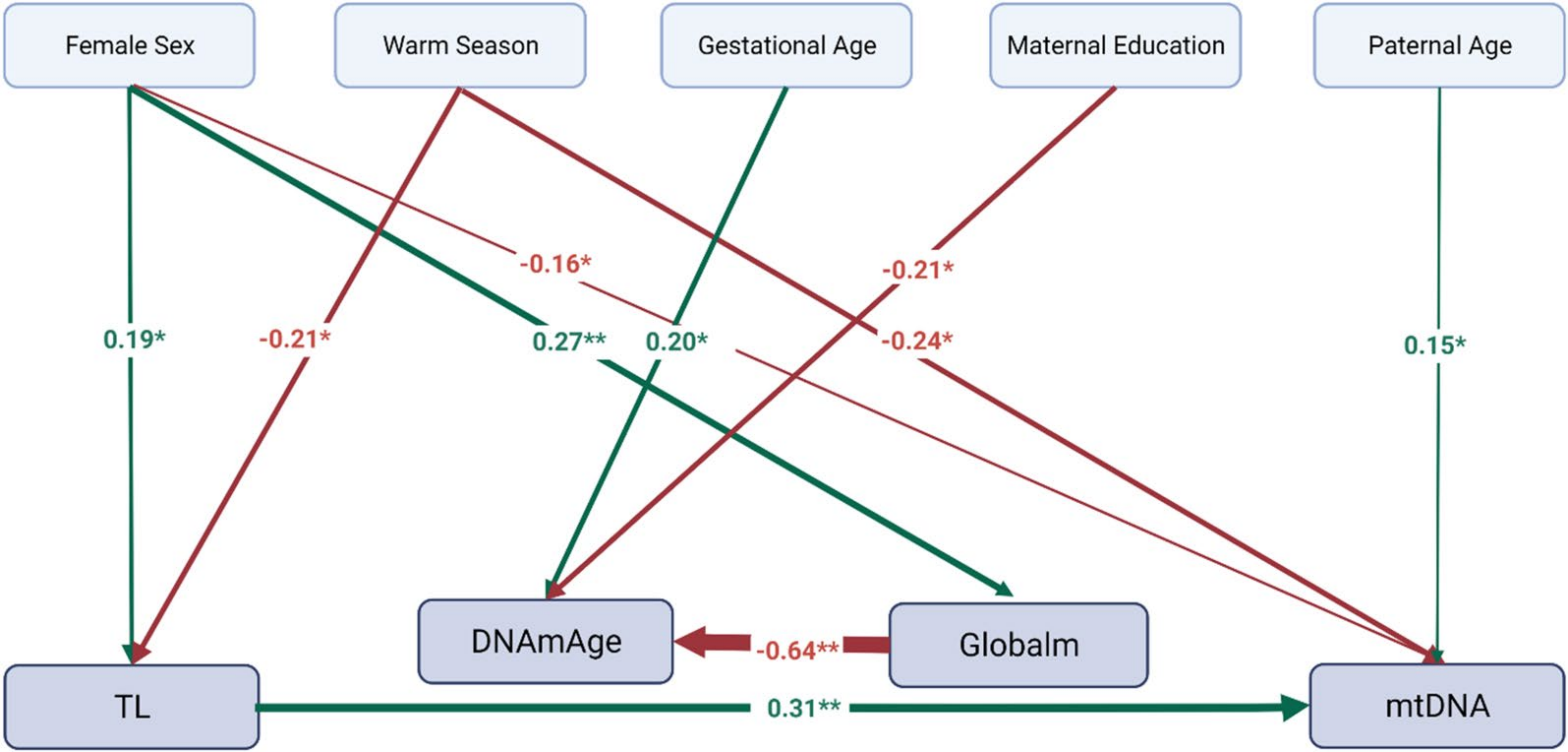
Graphical display of the path analysis model showing only the p < 0.05 significant standardized estimates for the multiple regression analyses with the four markers of biological age as endogenous variables (bottom). The analysis's exogenous variables comprised the other respective aging biomarkers, sex, gestational age, newborn ethnicity, birthweight, maternal smoking, maternal education, maternal early-pregnancy BMI, and parity. For TL and mtDNA content, additionally, white blood cell count, season of delivery and parental age, and for global methylation cell-type distribution according to Bakulski [51], were included as covariates. The coefficients in the figure were standardized, representing a 1 SD change in each exposure pathway. Red color stands for negative and green color for positive associations. The arrow's width indicates the degree of correlation, with wider arrows indicating higher correlation. Significant associations between cord blood cell composition and white blood cell count are not shown for the sake of clarity. DNAmAge = epigenetic age calculated according to Horvath [9]; Gestat.Age = gestational age in days; Globalm = global DNA methylation; TL = relative telomere length; mtDNA = mitochondrial DNA content; Warm Season = warm season (April 1 – September 30). *p < 0.05; **p < 0.001

The path analysis model representing the causal assumptions of the relationships between the aging biomarkers and *a priori* selected covariates showed a good overall fit (χ2 = 23.00, degrees of freedom = 27, p = 0.69; Root Mean Square Error of Approximation = 0.00, p = 0.963, Standardized Root Mean Square Residual = 0.034, Comparative Fit Index = 1.00). A graphical indication of the significant associations with standardized estimates is shown in Fig. 4.

Assuming all other variables were held constant, the strongest effect was shown for global methylation on DNAm age with a decrease of 0.64 standard deviations (SD) in DNAm age for each SD (0.01%) increase in global methylation (p < 0.001). The unstandardized coefficient translated to a decrease of 0.21 years of DNAm age for an IQR increase (0.011 units) of global methylation. For each SD increase in log_10_ transformed TL, the mean level of log_10_ transformed mtDNA content increased by 0.31 SD (p < 0.001) [for the unstandardized coefficients mtDNA content increased by 6.96% (95% CI: 4.97%, 8.95%) for a 10% increase in TL].

Concerning the covariates, season of delivery was associated with a decrease of 0.21 SD in log_10_ transformed TL and 0.24 SD in log_10_ transformed mtDNA content (p = 0.02 and p = 0.003, respectively) for the warmer half of the year compared to the cold half of the year. This corresponds for the unstandardized coefficients to a decrease of − 7.10% (95% CI: − 3.44%, − 10.63%) in TL and − 16.44% (95% CI: − 8.64%, − 23.58%) in mtDNA content respectively. Furthermore, an increase of one SD in gestational age (1.54 years) was associated with an increase of 0.20 SD in DNAm age (p = 0.017), indicating a 1.25 weeks higher DNAm age for each additional week of gestation. Newborns girls had on average 5.68% (95% CI: 2.95%–8.48%) longer telomeres, corresponding to an increase of 0.19 SD in mean log_10_ transformed cord blood TL (p < 0.001), or unstandardized to an increase of 6.4% cord blood TL (95% CI: 4.38–8.42). Additionally, female sex was associated with a 0.3 unit higher global methylation (0.27 SD, p < 0.001), and a 11.49% (95% CI: − 9.43%, − 13.55%) lower mtDNA content (0.16 SD, p = 0.016). Moving from a lower maternal educational level to a higher one was associated with a decrease of 0.21 SD (p = 0.014) in DNAm age or a decrease of about 2.77 weeks in DNAm age for the unstandardized coefficient. An increase of one SD (5.34 years) in paternal age was linked to an increase of 0.15 SD (p = 0.027) in log_10_ transformed mtDNA content, or for the unstandardized coefficient to an increase of 1.20% mtDNA content for an increase in one year of paternal age.

## Discussion

Over the years, different biological markers have been developed to track chronological age and predict the onset of various age-related diseases and risks of different lifestyle factors in adults. The investigation of newborns may shed light on mechanisms that could explain differences in disease susceptibility later in life through fetal programming. Here, we provide insight into molecular aging signatures at the start of life and their interrelations, showing that cord blood DNAm age is inversely associated with global methylation and mtDNA content but not with newborn telomere length. Furthermore, we provide evidence that the telomere-mitochondrial aging axis is already connected from early life onwards.

### Interrelationships between biomarkers of aging

With this study on newborns, we demonstrate an absence of correlation between TL and epigenetic age consistent with findings in adults [31] (Fig. 1 and Additional file 1: Table S1). A cross-sectional study including 800 middle-aged persons found no significant correlation between blood TL and DNAm age (*r* = − 0.05, *p* = 0.17) [32]. Furthermore, in 773 participants (mean chronological age = 69.68) of the LipidCardio Study no significant association between TL and DNAm age (β = 3.00, p = 0.18) was demonstrated [27]. Besides, the dynamics of both factors have also been shown to change throughout life. Telomere attrition is higher in young children when environmental influences exert the most impact on inter-individual variation in telomere length, which is subsequently preserved throughout life [21]. Likewise, the DNA methylation-based biological clock "ticks" differently over the life course. The Horvath clock shows a non-linear rate of the clock ticking faster than chronological aging during childhood and adolescence and a linear association with chronological years during adulthood [53]. In older people, an increase in DNAm age occurs even at a slower rate than chronological age [54]. Findings concerning the correlation between blood TL and DNAm age in adult and elderly populations are, therefore, not automatically transferrable to early childhood. However, this is the period that is presumably the most sensitive to environmental influences and sets the base for later life [21]. Our findings are important as they indicate that cord blood TL and DNAm age not only relate to different pathways/mechanisms of biological aging in adults but also in neonates for the period of fetal programming. Although telomere length and the epigenetic clock in newborns indicate different aging measures, this does not mean that DNA methylation is not linked with TL. Distinct epigenetic signatures were identified, and epigenetic regulation of newborn TL was reported previously [55] involving CpGs distinct from the CpG-sets used to predict biological age by epigenetic age clocks. Moreover, variants in Telomerase Reverse Transcriptase (*TERT*) gene on chromosome 5, associated with Horvath DNAm age derived intrinsic epigenetic age acceleration, were also found to be associated with longer telomeres, indicating that hTERT expression is required for DNAm aging in human primary fibroblast [56].

The finding of a correlation between mtDNA content and TL in cord blood is in line with previous findings of a positive association in 613 cord blood samples of the ENVIR*ON*AGE cohort, showing a 5.22% (95% CI: 3.26 to 7.22; p < 0.0001) higher mtDNA content for a 10% increase in TL [57]. Furthermore, also in healthy adults (r = 0.120, p < 0.001) [35], older women (r = 0.39, p < 0.0001) [28] and 166 non-smoking elderly (r = 0.23, p = 0.0047) [29] TL and mtDNA content were positively associated, conform with the mitochondrial-telomere axis of aging [23, 24, 57].

We also detected a correlation between global DNA methylation and three functional gene regions (promoter, gene body, intergenic) with DNAm age. In adults, no evidence for such an association was found in a previous pooled analysis of 479 individuals from the Australian Mammographic Density Twins and Sisters and 3354 individuals from the Melbourne Collaborative Cohort Study (r = 0.01, p > 0.19) [36]. This could be explained by the different dynamics of global methylation and DNAm age in newborns and adults. Global levels of DNA methylation increase over the first few years of life [58], remain relatively stable during adulthood, and then decrease beginning in late adulthood [59].

Another finding of our study is the inverse correlation between DNAm age and mtDNA content in cord blood. This finding is corroborated by a recent study in 812 older males of the Veteran Affairs Normative Aging Study, where the mtDNA copy number was negatively associated with cross-sectional, though not with prospective measures of DNAm age (p = 0.03 and p = 0.33 respectively) [30] (Fig. 1). In contrast, in a case–control study, DNAm age-derived AA and mtDNA content were not significantly correlated in the entire group of patients with bipolar disorder (BD), their siblings, and healthy age-matched controls (r = 0.038, p = 0.780), though positively correlated within the older (33-51 years) group of BD patients (r = 0.697, p < 0.001) [37] (Fig. 1). The different directions of correlation in our study of healthy newborns and the case–control study of BD patients may be partially explained by underlying differences in the biochemical mechanisms and etymology of BD.

### Associations between the aging biomarkers and covariates

The results of the path analysis confirmed previous findings in cord blood that at delivery, female sex is positively associated with telomere length [5, 21, 22, 60, 61] and global methylation [62]. In another study applying pyrosequencing of LINE-1 as a proxy of global methylation [63], an inverse association with the female sex was reported. Also, our finding of a negative association between female sex and cord blood mtDNA content is in line with previous findings. A study of the FLEHS III birth cohort in Flanders, Belgium, also found a trend for newborn girls having, on average, 5.96% less mtDNA content than boys (p = 0.08) [64].

Furthermore, delivery during the warm season was associated with shorter telomeres and less mtDNA content, which is in line with earlier findings of a longitudinal study including 2,827 residents of Costa Rica with a baseline age of 60 and older of longer telomeres in blood collected in October-December [65]. Moreover, a recent study in the ENVIR*ON*AGE birth cohort found prenatal temperature exposure above a certain threshold associated with shorter cord blood TL [66]. We also observed a negative association between the warm season and mtDNA content, which is in contrast to findings in the same cohort on placental tissue mtDNA content, reporting a negative association between mtDNA content and the cold season (β = -0.243 ± 0.040, p < 0.0001) [48]. A possible explanation for the discordance in the mtDNA content of the two tissues may originate from their different biological function, which has been postulated previously [67].

Our finding of an inverse association between maternal educational level with DNAm age is in accordance with previous findings on the relation between cord-blood DNAm age and maternal socioeconomic status (SES), another measure of maternal educational attainment. [68]. Furthermore, the association between paternal age at birth and mtDNA content corroborates an earlier report in adults [69].

With regard to the causal assumptions in the path analysis, different possibilities concerning the direction of effect between the aging biomarkers are reasonable. On the one hand, telomere-dependent growth arrest is associated with increased mitochondrial dysfunction [70] through suppression of *PGC-1α* and *PGC-1β* promoters, impairing mitochondrial biogenesis and function [71]; on the other hand, mitochondrial dysfunction leads to telomere attrition and genomic instability via the increase in oxidative stress [70, 72]. Furthermore, global hypomethylation is linked with biological aging via the loss of constitutive heterochromatin integrity, a hallmark of aging in eukaryotes leading to global 5mC with increased genetic instability as a result [17, 73]. The generation of important co-substrates required for histone phosphorylation, acetylation and deacetylation processes, such as adenosine triphosphate (ATP), acetyl CoA, flavin adenine dinucleotide, and nicotinamide adenine dinucleotide depends again on mitochondrial activity [74]. In a study of the ENVIR*ON*AGE cohort investigating the relationship between epigenome-wide methylation with cord blood insulin and mtDNA content, several pathways and differentially methylated regions (DMRs) also pointed in the direction of histone modification as one of the underlying mechanisms connecting these factors [75]. Dysfunctions in mitochondrial activity represented by alterations in mtDNA content may, therefore, have direct effects on global DNA methylation profiles [76, 77]. These molecular changes are not limited to the postnatal period, as environmental influences during pregnancy have been shown to already determine the genome methylation status and telomere length at birth [78, 79].

### Clinical relevance

The significance of cord blood aging biomarkers lies in their predictive value for age-related NCD long before actual health effects become visible. We recently found that telomere length in childhood and early adulthood is highly determined by cord blood telomere length [21]. Therefore, observed health and disease conditions related to shorter telomeres, may find to some extent their onset at birth. Understanding the interrelationships and determinants of aging biomarkers is essential from a prevention point of view, as these insights enable the early recognition of individuals with increased risks and the development of personalized treatment plans.

Telomere length at birth determines the natural life span [80], and it has been estimated, that at an age of 40 years, the difference in life expectancy between people with an 1 SD shorter or longer TL than the population mean amounts to 2.5 years [81]. Studies directly investigating the predictive power of aging biomarkers at birth with life-span and age-related disease in adults and older people are lacking. However, their relationship with childhood health measures may act as a surrogate for their association with health outcomes later in life, as previous studies demonstrated a link between health outcomes in childhood and adulthood [82–84].

**Fig. 5:**
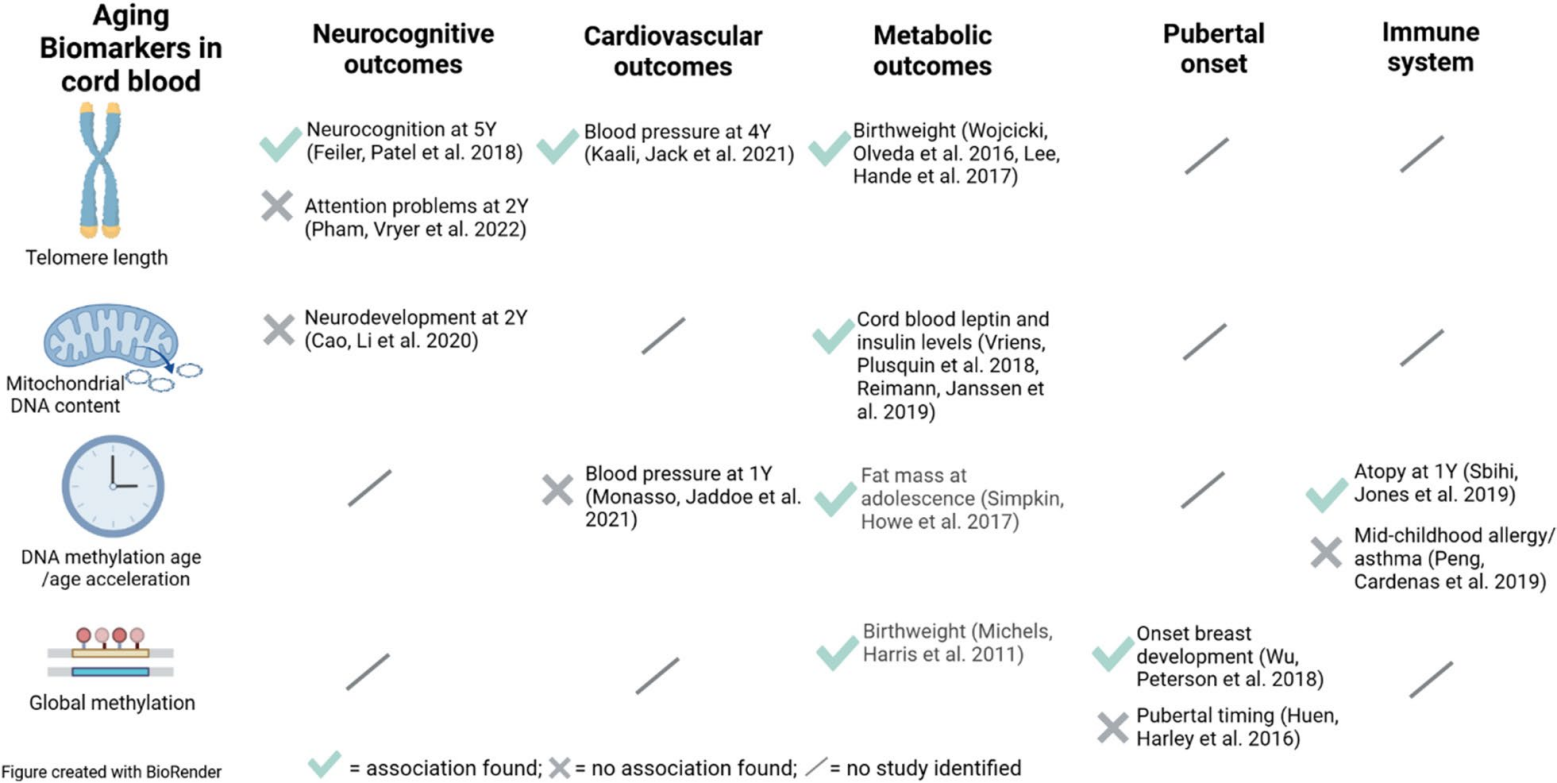
Associations between the cord blood aging biomarkers investigated in this study and health outcomes at birth and later in life were found in previous studies. A green check mark indicates that the study reported an association, and a grey cross indicates the lack of an association. A slash indicates that no study investigating the relationship with the health outcome was identified. Y = years of age

Cord blood aging biomarkers have been linked with deviating health measures in infancy and childhood, showing associations with neurocognitive [85–87], cardiovascular [88, 89] and metabolic outcomes [60, 75, 90–93], pubertal onset [94, 95] and the immune system [96, 97] (Fig. 5). In this context we recently demonstrated that telomere length at birth was significantly related to childhood diastolic blood pressure at the age of four [98]. Despite the lack of studies in many fields of age-related health measures, the fact that metabolic outcomes are related to all four cord blood aging biomarkers (Fig. 5) stresses the importance of alterations in the childhood metabolism linking cord blood aging biomarkers with adverse health outcomes at old age. Deviations from childhood health measures can be predictive of adverse health outcomes in adulthood and old age for example lower birthweight or weight at the age of 1 year have been associated with later life cardiovascular disease [99–101], diabetes type II [102], and frailty [103]. Furthermore, childhood BP was predictive of cardiovascular health in adulthood [104], pubertal timing was associated with multiple morbidities and lifespan in men [105], and infant eczema predicted adult asthma [106].

Concerning the determinants of aging biomarkers, identified in this study, their effect could translate to an altered disease susceptibility later in life. Children born in the warmer half of the year have on average shorter telomeres and lower mitochondrial DNA content, predisposing them to adverse infancy and childhood health measures such as lower birthweight, lower neurocognitive performance and higher blood pressure. On a population level, this could in turn result in a higher susceptibility to cardiovascular disease, diabetes type II, and frailty, therefore lowering life expectancy at old age.

### Strengths and limitations

Our study has several strengths and limitations. This is, to our knowledge, the first study to investigate the correlation between TL, DNAm age, global methylation, and mtDNA content in neonates, which is essential to disentangle their relationships in this crucial developmental stage. We also used data from a birth cohort that reflects the physiological ranges of the measured variables. On the other hand, the sample size of 190 newborns may have been too small to sustain significant results. Furthermore, the analysis of epigenetic age was confined to the algorithm published by Horvath [9]; other algorithms like the Hannum predictor [107] or various “gestational clocks” [108, 109] were not investigated as our focus was on the aging processes. We did, however, include gestational age in the path analysis. Regarding the Horvath clock, age acceleration is often studied besides age methylation Our study could not access a potential age acceleration as newborns have the same chronological age. Furthermore, path analysis made it necessary to hypothesize about the direction of effects between the aging biomarkers. Based on previous literature findings, evidence for both possible directions could often be found, making it necessary to choose one direction despite plausible reasons for another assumption.

## Conclusions

As the rate of age-associated functional decline may already be determined before birth, the status of aging biomarkers in cord blood could have important implications for overall life expectancy and disease susceptibility later in life. DNAm age and TL were significantly correlated with mtDNA content in our study, yet no relationship was observed between TL and DNAm age. This suggests that both biomarkers capture different aspects of aging from birth onwards and underlines the importance of the directed use of these biomarkers in the future risk assessment and early prevention of age-related disease. Path analysis demonstrated that the associations between the different aging biomarkers persist in a complex structure of interrelationships and environmental factors that better approximate the biological background. Moreover, comparing the standardized path coefficients makes it possible to estimate the extent of susceptibility to different internal and external influences and confirms previous observations of sex-dependent differences and the importance of prenatal temperature exposure in aging.

## Supplementary Information

**Additional file 1: Table S1.** Overview over the identified studies investigating the interrelationships between the aging biomarkers DNA methylation age (DNAm age), global genome-wide DNA methylation (global methylation), telomere length (TL) and mitochondrial DNA content (mtDNA content).

**Additional file 2: Text S1.** Modified quantitative real-time PCR (qPCR) protocol for the determination of average relative telomere length and mtDNA content

**Additional file 3: Table S2.** Cycling conditions Real Time PCR System 7900 HT. **Table S3.** Cycling conditions Real Time PCR System 7900 HT for the 36B4 gene. **Table S4.** Cycling conditions Fast Real Time PCR System 7900 HT for the mitochondrial gene copy numbers and two single-copy nuclear control genes.

**Additional file 4: Figure S1.** Pearson correlations between aging biomarkers in the subset of n = 92 girls

**Additional file 5: Figure S2.** Pearson correlations between aging biomarkers in the subset of n = 98 boys

## Data Availability

The data presented in this study are available on reasonable request from the corresponding authors. The data are not publicly available due to privacy restrictions.

## Abbreviations

5mC: 5-Methylcytosine
AA: Age acceleration
ATP: Adenosine triphosphate
BD: Bipolar disorder
BMI: Body mass index
DMRs: Differentially methylated regions
DNAm age: DNA methylation age
DNMT1: DNA (cytosine-5)-methyltransferase 1
global methylation: Global genome-wide DNA methylation
IQR: Interquartile range
mtDNA content: Mitochondrial DNA content
NCD: Non-communicable diseases
nDNA: Nuclear DNA
SD: Standard deviations
SEM: Structural equation modelling
T/S: Telomere/ single copy gene ratio
TL: Telomere length

## References

[1] Peel N, McClure R, Bartlett H (2005). Behavioral determinants of healthy aging 1. Am J Prev Med.

[2] Brooks-Wilson AR (2013). Genetics of healthy aging and longevity. Hum Genet.

[3] Vaiserman AM (2014). Early-life nutritional programming of longevity. J Dev Orig Health Dis.

[4] Martens DS, Cox B, Janssen BG, Clemente DBP, Gasparrini A, Vanpoucke C (2017). Prenatal air pollution and newborns’ predisposition to accelerated biological aging. JAMA Pediatr.

[5] Martens DS, Plusquin M, Gyselaers W, De Vivo I, Nawrot TS (2016). Maternal pre-pregnancy body mass index and newborn telomere length. BMC Med.

[6] Bijnens EM, Zeegers MP, Derom C, Martens DS, Gielen M, Hageman GJ (2017). Telomere tracking from birth to adulthood and residential traffic exposure. BMC Med.

[7] Barker DJ (1995). Fetal origins of coronary heart disease. BMJ.

[8] Muezzinler A, Zaineddin AK, Brenner H (2013). A systematic review of leukocyte telomere length and age in adults. Ageing Res Rev.

[9] Horvath S (2013). DNA methylation age of human tissues and cell types. Genome Biol.

[10] Horvath S, Erhart W, Brosch M, Ammerpohl O, von Schönfels W, Ahrens M (2014). Obesity accelerates epigenetic aging of human liver. Proc Natl Acad Sci.

[11] Perna L, Zhang Y, Mons U, Holleczek B, Saum K-U, Brenner H (2016). Epigenetic age acceleration predicts cancer, cardiovascular, and all-cause mortality in a German case cohort. Clin Epigenetics.

[12] Zheng Y, Joyce BT, Colicino E, Liu L, Zhang W, Dai Q (2016). Blood epigenetic age may predict cancer incidence and mortality. EBioMedicine.

[13] Ambatipudi S, Horvath S, Perrier F, Cuenin C, Hernandez-Vargas H, Le Calvez-Kelm F (2017). DNA methylome analysis identifies accelerated epigenetic ageing associated with postmenopausal breast cancer susceptibility. Eur J Cancer.

[14] Chen BH, Marioni RE, Colicino E, Peters MJ, Ward-Caviness CK, Tsai PC (2016). DNA methylation-based measures of biological age: meta-analysis predicting time to death. Aging.

[15] Marioni RE, Shah S, McRae AF, Chen BH, Colicino E, Harris SE (2015). DNA methylation age of blood predicts all-cause mortality in later life. Genome Biol.

[16] Oberdoerffer P, Michan S, McVay M, Mostoslavsky R, Vann J, Park S-K (2008). SIRT1 redistribution on chromatin promotes genomic stability but alters gene expression during aging. Cell.

[17] Ciccarone F, Tagliatesta S, Caiafa P, Zampieri M (2018). DNA methylation dynamics in aging: how far are we from understanding the mechanisms?. Mech Ageing Dev.

[18] Casillas MA, Lopatina N, Andrews LG, Tollefsbol TO (2003). Transcriptional control of the DNA methyltransferases is altered in aging and neoplastically-transformed human fibroblasts. Mol Cell Biochem.

[19] Mengel-From J, Thinggaard M, Dalgård C, Kyvik KO, Christensen K, Christiansen L (2014). Mitochondrial DNA copy number in peripheral blood cells declines with age and is associated with general health among elderly. Hum Genet.

[20] Zhang R, Wang Y, Ye K, Picard M, Gu Z (2017). Independent impacts of aging on mitochondrial DNA quantity and quality in humans. BMC Genomics.

[21] Martens DS, Van Der Stukken C, Derom C, Thiery E, Bijnens EM, Nawrot TS (2021). Newborn telomere length predicts later life telomere length: tracking telomere length from birth to child- and adulthood. EBioMedicine.

[22] Factor-Litvak P, Susser E, Kezios K, McKeague I, Kark JD, Hoffman M (2016). Leukocyte telomere length in newborns: implications for the role of telomeres in human disease. Pediatrics.

[23] Martens DS, Nawrot TS (2016). Air pollution stress and the aging phenotype: the telomere connection. Curr Environ Health Rep.

[24] Sahin E, DePinho RA (2012). Axis of ageing: telomeres, p53 and mitochondria. Nat Rev Mol Cell Biol.

[25] Sahin E, Depinho RA (2010). Linking functional decline of telomeres, mitochondria and stem cells during ageing. Nature.

[26] Judge S, Jang YM, Smith A, Hagen T, Leeuwenburgh C (2005). Age-associated increases in oxidative stress and antioxidant enzyme activities in cardiac interfibrillar mitochondria: implications for the mitochondrial theory of aging. FASEB J.

[27] Banszerus VL, Vetter VM, Salewsky B, Konig M, Demuth I (2019). Exploring the relationship of relative telomere length and the epigenetic clock in the lipidcardio cohort. Int J Mol Sci.

[28] Kim JH, Kim HK, Ko JH, Bang H, Lee DC (2013). The relationship between leukocyte mitochondrial DNA copy number and telomere length in community-dwelling elderly women. PLoS ONE.

[29] Pieters N, Janssen B, Valeri L, Cox B, Cuypers A, Dewitte H (2015). Molecular responses in the telomere-mitochondrial axis of ageing in the elderly: a candidate gene approach. Mech Ageing Dev.

[30] Dolcini J, Wu H, Nwanaji-Enwerem JC, Kiomourtozlogu MA, Cayir A, Sanchez-Guerra M (2020). Mitochondria and aging in older individuals: an analysis of DNA methylation age metrics, leukocyte telomere length, and mitochondrial DNA copy number in the VA normative aging study. Aging.

[31] Marioni RE, Harris SE, Shah S, McRae AF, von Zglinicki T, Martin-Ruiz C (2016). The epigenetic clock and telomere length are independently associated with chronological age and mortality. Int J Epidemiol.

[32] Belsky DW, Moffitt TE, Cohen AA, Corcoran DL, Levine ME, Prinz JA (2018). Eleven telomere, epigenetic clock, and biomarker-composite quantifications of biological aging: do they measure the same thing?. Am J Epidemiol.

[33] Vetter VM, Meyer A, Karbasiyan M, Steinhagen-Thiessen E, Hopfenmuller W, Demuth I (2019). Epigenetic clock and relative telomere length represent largely different aspects of aging in the berlin aging study II (BASE-II). J Gerontol A Biol Sci Med Sci.

[34] Li X, Ploner A, Wang Y, Magnusson PK, Reynolds C, Finkel D (2020). Longitudinal trajectories, correlations and mortality associations of nine biological ages across 20-years follow-up. Elife.

[35] Tyrka AR, Carpenter LL, Kao HT, Porton B, Philip NS, Ridout SJ (2015). Association of telomere length and mitochondrial DNA copy number in a community sample of healthy adults. Exp Gerontol.

[36] Chen M, Wong EM, Nguyen TL, Dite GS, Stone J, Dugué P-A (2019). DNA methylation-based biological age, genome-wide average DNA methylation, and conventional breast cancer risk factors. Sci Rep.

[37] Fries GR, Bauer IE, Scaini G, Wu M-J, Kazimi IF, Valvassori SS (2017). Accelerated epigenetic aging and mitochondrial DNA copy number in bipolar disorder. Transl Psychiatry.

[38] Vriens A, Nawrot TS, Janssen BG, Baeyens W, Bruckers L, Covaci A (2019). Exposure to environmental pollutants and their association with biomarkers of aging: a multipollutant approach. Environ Sci Technol.

[39] Alegría-Torres JA, Velázquez-Villafaña M, López-Gutiérrez JM, Chagoyán-Martínez MM, Rocha-Amador DO, Costilla-Salazar R (2016). Association of leukocyte telomere length and mitochondrial dna copy number in children from salamanca. Mexico Genet Test Mol Biomarkers.

[40] Hautekiet P, Nawrot TS, Janssen BG, Martens DS, De Clercq EM, Dadvand P (2021). Child buccal telomere length and mitochondrial DNA content as biomolecular markers of ageing in association with air pollution. Environ Int.

[41] Cerveira de Baumont A, Hoffmann MS, Bortoluzzi A, Fries GR, Lavandoski P, Grun LK (2021). Telomere length and epigenetic age acceleration in adolescents with anxiety disorders. Sci Rep.

[42] Janssen BG, Madlhoum N, Gyselaers W, Bijnens E, Clemente DB, Cox B (2017). Cohort profile: the environmental influence on early AGEing (ENVIRONAGE): a birth cohort study. Int J Epidemiol.

[43] World Medical Association (2013). Declaration of Helsinki: ethical principles for medical research involving human subjects. JAMA.

[44] Vineis P, Chadeau-Hyam M, Gmuender H, Gulliver J, Herceg Z, Kleinjans J (2017). The exposome in practice: design of the EXPOsOMICS project. Int J Hyg Environ Health.

[45] Bibikova M, Barnes B, Tsan C, Ho V, Klotzle B, Le JM (2011). High density DNA methylation array with single CpG site resolution. Genomics.

[46] Aryee MJ, Jaffe AE, Corrada-Bravo H, Ladd-Acosta C, Feinberg AP, Hansen KD (2014). Minfi: a flexible and comprehensive bioconductor package for the analysis of Infinium DNA methylation microarrays. Bioinformatics.

[47] Cawthon RM (2009). Telomere length measurement by a novel monochrome multiplex quantitative PCR method. Nucleic Acids Res.

[48] Janssen BG, Munters E, Pieters N, Smeets K, Cox B, Cuypers A (2012). Placental mitochondrial DNA content and particulate air pollution during in utero life. Environ Health Perspect.

[49] TELOMERE RESEARCH NETWORK. Study Design & analysis 2021 [Available from: https://trn.tulane.edu/resources/study-design-analysis/.

[50] Pelegí-Sisó D, de Prado P, Ronkainen J, Bustamante M, González JR (2020). methylclock: a Bioconductor package to estimate DNA methylation age. Bioinformatics.

[51] Bakulski KM, Feinberg JI, Andrews SV, Yang J, Brown SSLM (2016). DNA methylation of cord blood cell types: applications for mixed cell birth studies. Epigenetics.

[52] Rosseel Y (2011). lavaan an r package for structural equation modeling. J Stat Softw.

[53] Bell CG, Lowe R, Adams PD, Baccarelli AA, Beck S, Bell JT (2019). DNA methylation aging clocks: challenges and recommendations. Genome Biol.

[54] Marioni RE, Suderman M, Chen BH, Horvath S, Bandinelli S, Morris T (2018). Tracking the epigenetic clock across the human life course: a meta-analysis of longitudinal cohort data. J Gerontol Ser A.

[55] Wang C, Nawrot TS, Van Der Stukken C, Tylus D, Sleurs H, Peusens M (2021). Different epigenetic signatures of newborn telomere length and telomere attrition rate in early life. Aging.

[56] Lu AT, Xue L, Salfati EL, Chen BH, Ferrucci L, Levy D (2018). GWAS of epigenetic aging rates in blood reveals a critical role for TERT. Nat Commun.

[57] Van Der Stukken C, Nawrot TS, Alfano R, Wang C, Langie SAS, Plusquin M (2022). The telomere-mitochondrial axis of aging in newborns. Aging.

[58] Herbstman JB, Wang S, Perera FP, Lederman SA, Vishnevetsky J, Rundle AG (2013). Predictors and consequences of global DNA methylation in cord blood and at three years. PLoS ONE.

[59] Jones MJ, Goodman SJ, Kobor MS (2015). DNA methylation and healthy human aging. Aging Cell.

[60] Wojcicki JM, Olveda R, Heyman MB, Elwan D, Lin J, Blackburn E (2016). Cord blood telomere length in latino infants: relation with maternal education and infant sex. J Perinatol.

[61] Liu H, Zhou G, Chen Q, Ouyang F, Little J, Zhang J (2017). Impact of dehydroepiandrosterone sulfate on newborn leukocyte telomere length. Sci Rep.

[62] Yousefi P, Huen K, Davé V, Barcellos L, Eskenazi B, Holland N (2015). Sex differences in DNA methylation assessed by 450 K beadchip in newborns. BMC Genomics.

[63] Pilsner JR, Hall MN, Liu X, Ilievski V, Slavkovich V, Levy D (2012). Influence of prenatal arsenic exposure and newborn sex on global methylation of cord blood DNA. PLoS ONE.

[64] Vriens A, Nawrot TS, Baeyens W, Den Hond E, Bruckers L, Covaci A (2017). Neonatal exposure to environmental pollutants and placental mitochondrial DNA content: A multi-pollutant approach. Environ Int.

[65] Rosero-Bixby L, Rehkopf DH, Dow WH, Lin J, Epel ES, Azofeifa J (2019). Correlates of longitudinal leukocyte telomere length in the costa rican longevity study of healthy aging (CRELES) on the importance of DNA collection and storage procedures. PLoS ONE.

[66] Martens DS, Plusquin M, Cox B, Nawrot TS (2019). Early biological aging and fetal exposure to high and low ambient temperature: a birth cohort study. Environ Health Perspect.

[67] Gemma C, Sookoian S, Alvarinas J, Garcia SI, Quintana L, Kanevsky D (2006). Mitochondrial DNA depletion in small- and large-for-gestational-age newborns. Obesity.

[68] Javed R, Chen W, Lin F, Liang H (2016). Infant’s DNA methylation age at birth and epigenetic aging accelerators. Biomed Res Int.

[69] Mengel-From J, Svane AM, Pertoldi C, Nygaard Kristensen T, Loeschcke V, Skytthe A (2019). Advanced parental age at conception and sex affects mitochondrial DNA copy number in human and fruit flies. J Gerontol Ser A.

[70] Passos JF, Saretzki G, von Zglinicki T (2007). DNA damage in telomeres and mitochondria during cellular senescence: is there a connection?. Nucleic Acids Res.

[71] Sahin E, Colla S, Liesa M, Moslehi J, Müller FL, Guo M (2011). Telomere dysfunction induces metabolic and mitochondrial compromise. Nature.

[72] Liu L, Trimarchi JR, Smith PJS, Keefe DL (2002). Mitochondrial dysfunction leads to telomere attrition and genomic instability. Aging Cell.

[73] Saenen ND, Martens DS, Neven KY, Alfano R, Bové H, Janssen BG (2019). Air pollution-induced placental alterations: an interplay of oxidative stress, epigenetics, and the aging phenotype?. Clin Epigenetics.

[74] Shaughnessy DT, McAllister K, Worth L, Haugen AC, Meyer JN, Domann FE (2014). Mitochondria, energetics, epigenetics, and cellular responses to stress. Environ Health Perspect.

[75] Reimann B, Janssen BG, Alfano R, Ghantous A, Espín-Pérez A, de Kok TM (2019). The Cord blood insulin and mitochondrial DNA content related methylome. Frontiers Genet.

[76] Bellizzi D, D'Aquila P, Giordano M, Montesanto A, Passarino G (2012). Global DNA methylation levels are modulated by mitochondrial DNA variants. Epigenomics.

[77] D'Aquila P, Passarino G (2015). Mitochondria in health, aging and diseases: the epigenetic perspective. Biogerontology.

[78] Aagaard-Tillery KM, Grove K, Bishop J, Ke X, Fu Q, McKnight R (2008). Developmental origins of disease and determinants of chromatin structure: maternal diet modifies the primate fetal epigenome. J Mol Endocrinol.

[79] Clemente DBP, Vrijheid M, Martens DS, Bustamante M, Chatzi L, Danileviciute A (2019). Prenatal and childhood traffic-related air pollution exposure and telomere length in european children: the HELIX project. Environ Health Perspect.

[80] Heidinger BJ, Blount JD, Boner W, Griffiths K, Metcalfe NB, Monaghan P (2012). Telomere length in early life predicts lifespan. Proc Natl Acad Sci USA.

[81] Codd V, Wang Q, Allara E, Musicha C, Kaptoge S, Stoma S (2021). Polygenic basis and biomedical consequences of telomere length variation. Nat Genet.

[82] Blackwell DL, Hayward MD, Crimmins EM (2001). Does childhood health affect chronic morbidity in later life?. Social Sci Med.

[83] Currie J, Stabile M, Manivong P, Roos LL (2010). Child health and young adult outcomes. J Hum Resour.

[84] Smith JP, Shen Y, Strauss J, Zhe Y, Zhao Y (2012). The effects of childhood health on adult health and SES in China. Econ Dev Cult Change.

[85] Feiler MO, Patel D, Li H, Meacham PJ, Watson GE, Shamlaye C (2018). The association between early-life relative telomere length and childhood neurodevelopment. Neurotoxicology.

[86] Pham C, Vryer R, O’Hely M, Mansell T, Burgner D, Collier F (2022). Shortened infant telomere length is associated with attention deficit/hyperactivity disorder symptoms in children at age two years: a birth cohort study. Int J Mol Sci.

[87] Cao X, Li J, Cheng L, Deng Y, Li Y, Yan Z (2020). The associations between prenatal exposure to polycyclic aromatic hydrocarbon metabolites, umbilical cord blood mitochondrial DNA copy number, and children’s neurobehavioral development. Environ Pollut.

[88] Kaali S, Jack D, Opoku-Mensah J, Bloomquist T, Aanaro J, Quinn A (2021). Prenatal household air pollution exposure cord blood mononuclear cell telomere length and age four blood pressure evidence from a ghanaian pregnancy cohort. Toxics.

[89] Monasso GS, Jaddoe VWV, Küpers LK, Felix JF (2021). Epigenetic age acceleration and cardiovascular outcomes in school-age children: the generation r study. Clin epigenet.

[90] Lee S-P, Hande P, Yeo GS, Tan E-C (2017). Correlation of cord blood telomere length with birth weight. BMC Res Notes.

[91] Vriens A, Plusquin M, Baeyens W, Bruckers L, Den Hond E, Loots I (2018). Cord blood leptin and insulin levels in association with mitochondrial DNA content. J Transl Med.

[92] Simpkin AJ, Howe LD, Tilling K, Gaunt TR, Lyttleton O, McArdle WL (2017). The epigenetic clock and physical development during childhood and adolescence: longitudinal analysis from a UK birth cohort. Int J Epidemiol.

[93] Michels KB, Harris HR, Barault L (2011). Birthweight, maternal weight trajectories and global DNA methylation of LINE-1 repetitive elements. PLoS ONE.

[94] Wu Y, Peterson KE, Sánchez BN, Dolinoy DC, Mercado-Garcia A, Téllez-Rojo MM (2018). Association of blood leukocyte DNA methylation at LINE-1 and growth-related candidate genes with pubertal onset and progression. Epigenetics.

[95] Huen K, Harley K, Kogut K, Rauch S, Eskenazi B, Holland N (2016). DNA methylation of LINE-1 and Alu repetitive elements in relation to sex hormones and pubertal timing in Mexican-American children. Pediatr Res.

[96] Sbihi H, Jones MJ, MacIsaac JL, Brauer M, Allen RW, Sears MR (2019). Prenatal exposure to traffic-related air pollution, the gestational epigenetic clock, and risk of early-life allergic sensitization. J Allergy Clin Immunol.

[97] Peng C, Cardenas A, Rifas-Shiman SL, Hivert MF, Gold DR, Platts-Mills TA (2019). Epigenetic age acceleration is associated with allergy and asthma in children in project viva. J Allergy Clin Immunol.

[98] Martens DS, Sleurs H, Dockx Y, Rasking L, Plusquin M, Nawrot TS. Association of newborn telomere length with blood pressure in childhood JAMA Network Open 2022 (in press). 10.1001/jamanetworkopen.2022.25521 PMC9356312 35930283

[99] Barker DJ, Winter PD, Osmond C, Margetts B, Simmonds SJ (1989). Weight in infancy and death from ischaemic heart disease. Lancet.

[100] Rich-Edwards JW, Stampfer MJ, Manson JE, Rosner B, Hankinson SE, Colditz GA (1997). Birth weight and risk of cardiovascular disease in a cohort of women followed up since 1976. BMJ (Clinical research ed).

[101] Fall CH, Vijayakumar M, Barker DJ, Osmond C, Duggleby S (1995). Weight in infancy and prevalence of coronary heart disease in adult life. BMJ (Clinical research ed).

[102] Rich-Edwards JW, Colditz GA, Stampfer MJ, Willett WC, Gillman MW, Hennekens CH (1999). Birthweight and the risk for type 2 diabetes mellitus in adult women. Ann Intern Med.

[103] Haapanen MJ, Perälä MM, Salonen MK, Kajantie E, Simonen M, Pohjolainen P (2018). Early life determinants of frailty in old age: the helsinki birth cohort study. Age Ageing.

[104] Yang L, Magnussen CG, Yang L, Bovet P, Xi B (2020). Elevated blood pressure in childhood or adolescence and cardiovascular outcomes in adulthood. Hypertension.

[105] Hollis B, Day FR, Busch AS, Thompson DJ, Soares ALG, Timmers PRHJ (2020). Genomic analysis of male puberty timing highlights shared genetic basis with hair colour and lifespan. Nat Commun.

[106] Abo-Zaid G, Sharpe RA, Fleming LE, Depledge M, Osborne NJ (2018). Association of infant eczema with childhood and adult asthma: analysis of data from the 1958 birth cohort study. Int J Environ Res Public Health.

[107] Hannum G, Guinney J, Zhao L, Zhang L, Hughes G, Sadda S (2013). Genome-wide methylation profiles reveal quantitative views of human aging rates. Mol Cell.

[108] Knight AK, Craig JM, Theda C, Bækvad-Hansen M, Bybjerg-Grauholm J, Hansen CS (2016). An epigenetic clock for gestational age at birth based on blood methylation data. Genome Biol.

[109] Bohlin J, Haberg SE, Magnus P, Reese SE, Gjessing HK, Magnus MC (2016). Prediction of gestational age based on genome-wide differentially methylated regions. Genome Biol.

