# Supplementary material for: Interrelationships and determinants of aging biomarkers in cord blood
Source: J Transl Med. 2022 Aug 9;20:353. doi: 10.1186/s12967-022-03541-1 (PMC9361565; doi:10.1186/s12967-022-03541-1)
Supplement: Supplementary file 1 — Additional file 1: Table S1. Overview over the identified studies investigating the interrelationships between the aging biomarkers DNA methylation age (DNAm age), global genome-wide DNA methylation (global methylation), telomere length (TL) and mitochondrial DNA content (mtDNA content). [file 12967_2022_3541_MOESM1_ESM.docx]

**Interrelationships and determinants of aging biomarkers in cord blood**

**Brigitte Reimann^1^, Dries S. Martens^1^, Congrong Wang^1^, Akram Ghantous^2^, Zdenko Herceg^2^, Michelle Plusquin^1^* and Tim S. Nawrot^1,3^**

^1^ Centre for Environmental Sciences, Hasselt University, Hasselt, Belgium

^2^ Epigenomics and Mechanisms Branch, International Agency for Research on Cancer (IARC),

Lyon, France

^3^ School of Public Health, Occupational and Environmental Medicine, KU Leuven, Leuven,

Belgium

***** Correspondence: [michelle.plusquin@uhasselt.be](mailto:michelle.plusquin@uhasselt.be)

**Table S1** Overview over the identified studies investigating the interrelationships between the aging biomarkers DNA methylation age (DNAm age), global genome-wide DNA methylation (global methylation), telomere length (TL) and mitochondrial DNA content (mtDNA content).

| **Cohort/country** | **Sample size** | **% Woman** | **Mean Age (SD)** | **Biomarkers** | **Main findings** | **Reference** |
| --- | --- | --- | --- | --- | --- | --- |
| **Old Age** | | | | | | |
| LipidCardio Study, Germany | n = 773 | 31.6% | 69.7 ± 11.01 | TL DNAm age, DNAm AA | No association between TL, DNAm age or DNAm AA | (Banszerus, Vetter et al. 2019) |
| Yonsei Aging Study, Korea | n = 129 | 100% | 73.74 (6.99) | TL, mtDNA content | Positive correlation between mtDNA and TL | (Kim, Kim et al. 2013) |
| Nonsmoking men and women aged 60-80 years, Belgium | n = 166 | 54% | 70.6 (4.7) | TL, mtDNA content | Positive correlation between mtDNA and TL | (Pieters, Janssen et al. 2015) |
| Veteran Affairs Normative Aging Study (NAS), U.S. | n = 812 | 0% | 72.4 (6.9) | Cross-sectional and prospective associations between mtDNA content, DNAm-Age, TL, DNAmPhenoAge and DNAm-GrimAge | Inverse association mtDNA content and DNAm age in cross-sectional design.  Inverse association between mtDNA content and prospective measures of TL. | (Dolcini, Wu et al. 2020) |
| Lothian Birth Cohorts of 1921 (LBC1921) and 1936 (LBC1936), Great Britain | LBC1921: n = 920 LBC1936n = 414 | / | LBC1921: ages 70, 73 and 76 years; LBC1936: ages 79, 87 and 90 years | TL and DNAm age | No correlations between epigenetic clock estimates and TL | (Marioni, Harris et al. 2016) |
|  | | | | | | |
| **Adults** | | | | | | |
| Dunedin Study, New Zealand | n = 1,037 | 48% | 38 | TL, three epigenetic-clocks, and three biomarker-composites | Low correlation between the epigenetic clocks, and TL | (Belsky, Moffitt et al. 2018) |
| Berlin Aging Study II consisting of a a.) young and an b.) old age group, Germany | a.) n = 424  b.) n = 1,471 | a.) 51.4%  b.) 49.4% | a.) 28.8 (3.1)  b.) 68.7 (3.7) | TL, DNAm age, DNAm AA | Inverse association between DNAm age and TL in adjusted linear regression analysis | (Vetter, Meyer et al. 2019) |
| Swedish Adoption/Twin Study of Aging (SATSA), Sweden | n = 845  (n = 288 with all biomarkers) | 59.5% | 63.6 (8.6) | TL, four DNAm ages, and three functional ages | Low correlation between TL and other markers | (Li, Ploner et al. 2020) |
| Healthy Adults from studies assessing adversity and depressive and anxiety disorder (pooled data), U.S. | n = 392 | 63% | 31.4 (11.2) | TL, mtDNA content | Positive correlation between mtDNA and TL in the entire sample and all subgroups | (Tyrka, Carpenter et al. 2015) |
| Australian Mammographic Density Twins and Sisters, Australia | n = 479 | 100% | 55.7 (8.0) | DNAm age based on three epigenetic clocks and global methylation based on average methylation beta-value | No correlation between DNAm age and global methylation | (Chen, Wong et al. 2019) |
| Patients with bipolar disorder, their siblings and healthy controls, U.S. | Patients: n = 22  Siblings: n = 16  Controls: n = 20 | Patients: 68%  Siblings: 63%  Controls: 60% | Patients: 33.95 (9.3);  Siblings: 39 (10.6);  Controls: 34.75 (10.0) | global DNA methylation, mtDNA content, TL and DNAm age | Correlation mtDNA content with DNAm AA in patients aged 33+. No correlation between TL with DNAm age or mtDNA content | (Fries, Bauer et al. 2017) |
| Flemish Environment and Health Study (FLEHS), Belgium | N = 175 | 48.6% | 58.3 (4.0) | TL, mtDNA content | Positive correlation between mtDNA and TL | (Vriens, Nawrot et al. 2019) |
|  | | | | | | |
| **Adolescents** | | | | | | |
| Healthy children and adolescents, Mexico | n = 98 | / | Range: 6-12 | mtDNA content, TL | Positive association between TL and mtDNA content | (Alegría-Torres, Velázquez-Villafaña et al. 2016) |
| Primary schoolchildren, Belgium | n = 197 | 48.7% | 10.3 (1.3) | Buccal mtDNA content and TL | No association between TL and mtDNA content | (Hautekiet, Nawrot et al. 2021) |
| Longitudinal study with 5-year follow-up of adolescents from public schools, Brazil | n = 234  n = 76 (follow-up) | 61% | 13.4 (2.37) at baseline | TL and DNAm AA | No correlation between TL and DNAm AA at baseline or follow-up | (Cerveira de Baumont, Hoffmann et al. 2021) |

**References:**

Alegría-Torres, J. A., M. Velázquez-Villafaña, J. M. López-Gutiérrez, M. M. Chagoyán-Martínez, D. O. Rocha-Amador, R. Costilla-Salazar and L. García-Torres (2016). "Association of Leukocyte Telomere Length and Mitochondrial DNA Copy Number in Children from Salamanca, Mexico." Genet Test Mol Biomarkers **20**(11): 654-659.

Banszerus, V. L., V. M. Vetter, B. Salewsky, M. Konig and I. Demuth (2019). "Exploring the Relationship of Relative Telomere Length and the Epigenetic Clock in the LipidCardio Cohort." Int J Mol Sci **20**(12).

Belsky, D. W., T. E. Moffitt, A. A. Cohen, D. L. Corcoran, M. E. Levine, J. A. Prinz, J. Schaefer, K. Sugden, B. Williams, R. Poulton and A. Caspi (2018). "Eleven Telomere, Epigenetic Clock, and Biomarker-Composite Quantifications of Biological Aging: Do They Measure the Same Thing?" Am J Epidemiol **187**(6): 1220-1230.

Cerveira de Baumont, A., M. S. Hoffmann, A. Bortoluzzi, G. R. Fries, P. Lavandoski, L. K. Grun, L. S. P. Guimarães, F. T. C. R. Guma, G. A. Salum, F. M. Barbé-Tuana and G. G. Manfro (2021). "Telomere length and epigenetic age acceleration in adolescents with anxiety disorders." Scientific Reports **11**(1): 7716.

Chen, M., E. M. Wong, T. L. Nguyen, G. S. Dite, J. Stone, P.-A. Dugué, G. G. Giles, M. C. Southey, R. L. Milne, J. L. Hopper and S. Li (2019). "DNA methylation-based biological age, genome-wide average DNA methylation, and conventional breast cancer risk factors." Scientific Reports **9**(1): 15055.

Dolcini, J., H. Wu, J. C. Nwanaji-Enwerem, M. A. Kiomourtozlogu, A. Cayir, M. Sanchez-Guerra, P. Vokonas, J. Schwarz and A. A. Baccarelli (2020). "Mitochondria and aging in older individuals: an analysis of DNA methylation age metrics, leukocyte telomere length, and mitochondrial DNA copy number in the VA normative aging study." Aging (Albany NY) **12**(3): 2070-2083.

Fries, G. R., I. E. Bauer, G. Scaini, M.-J. Wu, I. F. Kazimi, S. S. Valvassori, G. Zunta-Soares, C. Walss-Bass, J. C. Soares and J. Quevedo (2017). "Accelerated epigenetic aging and mitochondrial DNA copy number in bipolar disorder." Translational Psychiatry **7**(12): 1283.

Hautekiet, P., T. S. Nawrot, B. G. Janssen, D. S. Martens, E. M. De Clercq, P. Dadvand, M. Plusquin, E. M. Bijnens and N. D. Saenen (2021). "Child buccal telomere length and mitochondrial DNA content as biomolecular markers of ageing in association with air pollution." Environ Int **147**: 106332.

Kim, J. H., H. K. Kim, J. H. Ko, H. Bang and D. C. Lee (2013). "The relationship between leukocyte mitochondrial DNA copy number and telomere length in community-dwelling elderly women." PLoS One **8**(6): e67227.

Li, X., A. Ploner, Y. Wang, P. K. Magnusson, C. Reynolds, D. Finkel, N. L. Pedersen, J. Jylhävä and S. Hägg (2020). "Longitudinal trajectories, correlations and mortality associations of nine biological ages across 20-years follow-up." Elife **9**.

Marioni, R. E., S. E. Harris, S. Shah, A. F. McRae, T. von Zglinicki, C. Martin-Ruiz, N. R. Wray, P. M. Visscher and I. J. Deary (2016). "The epigenetic clock and telomere length are independently associated with chronological age and mortality." International Journal of Epidemiology **45**(2): 424-432.

Pieters, N., B. G. Janssen, L. Valeri, B. Cox, A. Cuypers, H. Dewitte, M. Plusquin, K. Smeets and T. S. Nawrot (2015). "Molecular responses in the telomere-mitochondrial axis of ageing in the elderly: a candidate gene approach." Mech Ageing Dev **145**: 51-57.

Tyrka, A. R., L. L. Carpenter, H. T. Kao, B. Porton, N. S. Philip, S. J. Ridout, K. K. Ridout and L. H. Price (2015). "Association of telomere length and mitochondrial DNA copy number in a community sample of healthy adults." Exp Gerontol **66**: 17-20.

Vetter, V. M., A. Meyer, M. Karbasiyan, E. Steinhagen-Thiessen, W. Hopfenmüller and I. Demuth (2019). "Epigenetic Clock and Relative Telomere Length Represent Largely Different Aspects of Aging in the Berlin Aging Study II (BASE-II)." J Gerontol A Biol Sci Med Sci **74**(1): 27-32.

Vriens, A., T. S. Nawrot, B. G. Janssen, W. Baeyens, L. Bruckers, A. Covaci, S. De Craemer, S. De Henauw, E. Den Hond, I. Loots, V. Nelen, T. Schettgen, G. Schoeters, D. S. Martens and M. Plusquin (2019). "Exposure to Environmental Pollutants and Their Association with Biomarkers of Aging: A Multipollutant Approach." Environ Sci Technol **53**(10): 5966-5976.
