# Supplementary material for: Interrelationships and determinants of aging biomarkers in cord blood
Source: J Transl Med. 2022 Aug 9;20:353. doi: 10.1186/s12967-022-03541-1 (PMC9361565; doi:10.1186/s12967-022-03541-1)
Supplement: Supplementary file 2 — Additional file 2: Text S1. Modified quantitative real-time PCR (qPCR) protocol for the determination of average relative telomere length and mtDNA content [file 12967_2022_3541_MOESM2_ESM.docx]

**Interrelationships and determinants of aging biomarkers in cord blood**

**Brigitte Reimann^1^, Dries S. Martens^1^, Congrong Wang^1^, Akram Ghantous^2^, Zdenko Herceg^2^, Michelle Plusquin^1^* and Tim S. Nawrot^1,3^**

^1^ Centre for Environmental Sciences, Hasselt University, Hasselt, Belgium

^2^ Epigenomics and Mechanisms Branch, International Agency for Research on Cancer (IARC),

Lyon, France

^3^ School of Public Health, Occupational and Environmental Medicine, KU Leuven, Leuven,

Belgium

***** Correspondence: [michelle.plusquin@uhasselt.be](mailto:michelle.plusquin@uhasselt.be)

**Text S1 Modified quantitative real-time PCR (qPCR) protocol for the determination of average relative telomere length and mtDNA content**

The ratio of the telomeric region to the single-copy gene *RPLP0* region (1) was determined by amplifying the telomeric region with the use of telomere specific primers (telg and telc). For the determination of the mtDNA content the ratio of the copy numbers retrieved from (i) mitochondrial forward primer of nucleotide 3212, (ii) reverse primer of nucleotide 3319 (MTF3212/R3319) and (iii) mitochondrial encoded NADH dehydrogenase 1 (*MT-ND1*)] to two single-copy nuclear control genes, [acidic ribosomal phosphoprotein P0 (*RPLP0*) and beta-actin (*ACTB)*], was determined. For the reactions 7.5 µL master mix, consisting of 5 µL/reaction Fast SYBR R Green I dye (Applied Biosystems), forward and reverse primer (0.3 µL/reaction each), and RNase free water (1.9 µL/reaction) were aliquoted into MicroAmp R Fast Optical 384-Well Reaction Plates. To each well with master mix 2.5 µL from one of the diluted DNA samples were then added, obtaining a final volume of 10 µL per reaction. To estimate reaction efficiency a 6-point serial dilution with a start concentration of 8ng/µl of pooled buffy coat DNA and a dilution factor of 1:3 were run in triplicate with every reaction plate. Possible DNA contaminations were controlled using a non-template control (NTCs). To account for inter-run variability six inter-run calibrators (IRCs), were used. At the end of each run melting curve analyses confirmed the reaction specificity and absence of primer-dimers. The assay precision was assessed by computing the interclass coefficient (ICC) and its 95% CI of triplicate measures (T/S ratios) (2). Both the inter-assay (based on 10 IRCs over 16 qPCR plates) and intra-assay ICC (based on all measures) was calculated using the on-line R script of the Telomere Research Network (3).

**References**

1. Cawthon RM. Telomere measurement by quantitative PCR. Nucleic acids research. 2002;30(10):e47.

2. TELOMERE RESEARCH NETWORK. Study Design & Analysis 2021 [Available from: <https://trn.tulane.edu/resources/study-design-analysis/>.

3. Eisenberg. D, Nettle. D, Verhulst. S. How to calculate the repeatability (ICC) of telomere length measures 2020 [Available from: <https://trn.tulane.edu/wp-content/uploads/sites/445/2020/10/How-to-calculate-repeatability.pdf>.
