# Supplementary material for: Interrelationships and determinants of aging biomarkers in cord blood
Source: J Transl Med. 2022 Aug 9;20:353. doi: 10.1186/s12967-022-03541-1 (PMC9361565; doi:10.1186/s12967-022-03541-1)
Supplement: Supplementary file 3 — Additional file 3: Table S2. Cycling conditions Real Time PCR System 7900 HT. Table S3. Cycling conditions Real Time PCR System 7900 HT for the 36B4 gene. Table S4. Cycling conditions Fast Real Time PCR System 7900 HT for the mitochondrial gene copy numbers and two single-copy nuclear control genes. [file 12967_2022_3541_MOESM3_ESM.docx]

**Interrelationships and determinants of aging biomarkers in cord blood**

**Brigitte Reimann^1^, Dries S. Martens^1^, Congrong Wang^1^, Akram Ghantous^2^, Zdenko Herceg^2^, Michelle Plusquin^1^* and Tim S. Nawrot^1,3^**

^1^ Centre for Environmental Sciences, Hasselt University, Hasselt, Belgium

^2^ Epigenomics and Mechanisms Branch, International Agency for Research on Cancer (IARC),

Lyon, France

^3^ School of Public Health, Occupational and Environmental Medicine, KU Leuven, Leuven,

Belgium

***** Correspondence: [michelle.plusquin@uhasselt.be](mailto:michelle.plusquin@uhasselt.be)

**Tables S2 – S4 Cycling conditions Real Time PCR System 7900 HT**

**Table S2**. Cycling conditions Real Time PCR System 7900 HT for the telomere repeats

| Duration and Temperature | Number of Cycles |
| --- | --- |
| 10 min at 95°C | **1 cycle** |
| 15 sec at 94°C | **2 cycles** |
| 2 min at 49°C |  |
| 15 sec at 94°C | **30 cycles** |
| 20 sec at 62°C |  |
| 1 min 20 sec at 74°C |  |
|  |  |

**Table S3.** Cycling conditions Real Time PCR System 7900 HT for the 36B4 gene

| Duration and Temperature | Number of Cycles |
| --- | --- |
| 10 min at 95°C | **1 cycle** |
| 15 sec at 95°C | **40 cycles** |
| 1 min 20 sec at 58°C |  |

**Table S4.** Cycling conditions Fast Real Time PCR System 7900 HT for the mitochondrial gene copy numbers and two single-copy nuclear control genes

| Duration and Temperature | Number of Cycles |
| --- | --- |
| 20 sec at 95°C |  |
| 1 sec at 95°C | **40 cycles** |
| 20 sec at 60°C |  |
