# Supplementary material for: Interrelationships and determinants of aging biomarkers in cord blood
Source: J Transl Med. 2022 Aug 9;20:353. doi: 10.1186/s12967-022-03541-1 (PMC9361565; doi:10.1186/s12967-022-03541-1)
Supplement: Supplementary file 4 — Additional file 4: Figure S1. Pearson correlations between aging biomarkers in the subset of n = 92 girls [file 12967_2022_3541_MOESM4_ESM.docx]

**Interrelationships and determinants of aging biomarkers in cord blood**

**Brigitte Reimann^1^, Dries S. Martens^1^, Congrong Wang^1^, Akram Ghantous^2^, Zdenko Herceg^2^, Michelle Plusquin^1^* and Tim S. Nawrot^1,3^**

^1^ Centre for Environmental Sciences, Hasselt University, Hasselt, Belgium

^2^ Epigenomics and Mechanisms Branch, International Agency for Research on Cancer (IARC),

Lyon, France

^3^ School of Public Health, Occupational and Environmental Medicine, KU Leuven, Leuven,

Belgium

***** Correspondence: [michelle.plusquin@uhasselt.be](mailto:michelle.plusquin@uhasselt.be)

**Figure S1 Pearson correlations between aging biomarkers in the subset of n = 92 girls**

**
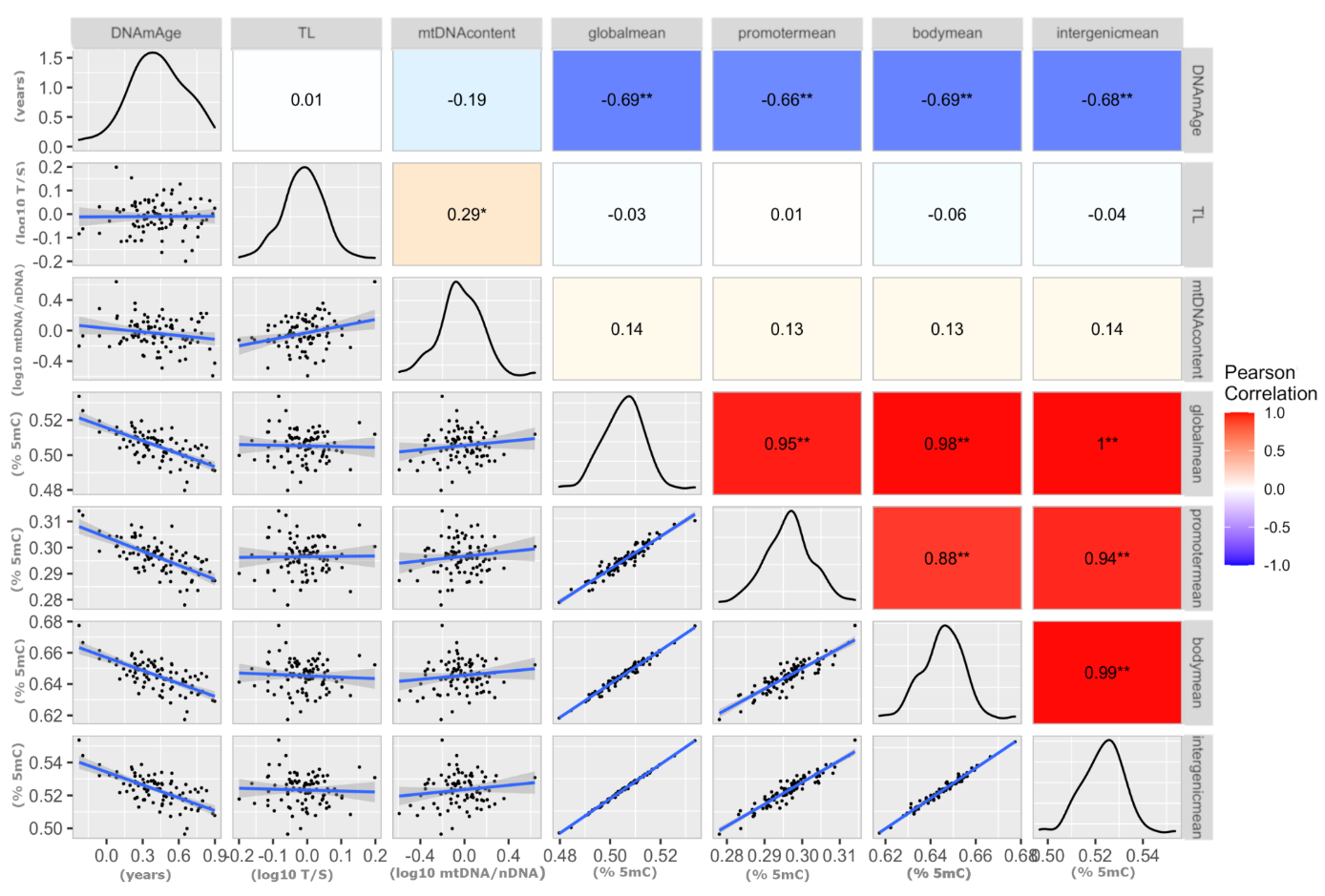
**

**Figure S1**

Pearson correlations in the subset of n = 92 girls, between: DNAm age = epigenetic age calculated according to Horvath (1), TL = relative telomere length, mtDNA content = relative mitochondrial DNA content, mean global DNA methylation = mean global DNA methylation, mean promoter methylation = mean methylation of the promoter gene-region, mean body methylation = mean methylation of the gene-body and mean methylation of the intergenic region. In the top right corner, the correlation coefficients, and in the bottom left corner scatterplots of the correlation with regression line and 0.95% CI are shown. On the diagonal density plots display the distribution of observations. 5mC = 5-methylcytosine; mtDNA = mitochondrial DNA; nDNA = nuclear DNA; T/S = telomere/ single copy gene ratio ** p <0.05; ** p <0.001*

**Reference**

1. Horvath S. DNA methylation age of human tissues and cell types. Genome biology. 2013;14(10):3156.
